# The Philadelphia Practice of Midwifery

**Published:** 1839-07

**Authors:** 


### Art. II.

1. *The Philadelphia Practice of Midwifery.* By Charles D. Meigs, m.d., Lecturer on Midwifery and of Diseases of Women and Children, &c.—*Philadelphia*, 1838. 8vo. pp. 370.

2. *Mémoires de Médecine et de Chirurgie pratique sur plusieurs Maladies et Accidens graves qui peuvent compliquer la Grossesse, la Parturition, et la Couche. Précédés d'un Compte-rendu analytique de Maladies observées à l'Hospice de la Charité de Lyon pendant un Exercice de sept Années.* Par le Docteur Martin, le jeune, &c.—*A Paris*, 1835. 8vo. pp. 462.

*Essays in Practical Medicine and Surgery, on various Diseases and important Circumstances complicating Pregnancy, Labour, and the Puerperal State, &c.* By Dr. Martin, jun., &c.

We have joined these two works together in one article, as well because the subjects of them are similar, as because they claim and shall receive from us the same kind of notice. In reviewing them, we shall only advert to such points of science or practice as are important, and on which right views are essential to the obstetrical student. In all our reviews of books, our main object is to be useful to the practitioner: mere criticism when divorced from its legitimate end, improvement, we hold in slight estimation; and so long as we keep this end in view, we care not how discursive we seem.

We shall treat of the two works included in the present article, in succession, beginning with that of Dr. Meigs.

I. The Philadelphia school of midwifery has for many years been looked upon with great respect by the obstetricians on this side of the Atlantic; the high name and professional standing of Dr. Dewees, his great experience, and, above all, his inestimable Compendious System of Midwifery and other valuable publications, have mainly contributed to this result. Dr. Meigs's volume is upon a much smaller scale than that of his distinguished countryman, but it contains much which is valuable and instructive to the student. The subjects are arranged in the usual order of works of this kind; beginning with the anatomy and physiology, and ending with the practical portion which comprehends the larger part of the book. Between these two main divisions, the author has introduced four chapters on Menstruation, Amenorrhœa, Dysmenorrhœa, and Leucorrhœa.

1. In enumerating the diameters of the pelvic brim and their dimensions, Dr. Meigs appears to have accidentally transferred the measurement of the transverse diameter to that of the oblique: he states that the transverse diameter of the brim measures from four and a half to five inches, and that the oblique diameters are five inches. The most correct measurements of the pelvis, show that the oblique diameters are at least half an inch shorter than the transverse in the skeleton state, and taken in the French inches, which are the standard most usually adopted on the continent, they run in the following order: antero-posterior diameter, four inches; oblique diameter, four inches and a half; transverse diameter, five inches. These are looked upon as the correct dimensions of the brim both in France and Germany, and, bearing in mind that the French inches are rather longer than our own, we shall come very nearly to the truth by making a small addition to each admeasurement. The numbers in French inches are peculiarly easy for the student to recollect, the antero-posterior, the oblique, and transverse diameters of the brim differing from each other by a successive increase of half an inch. It is to be regretted that the reader has not been made aware of this fact; the discrepancy in the admeasurements of different authors would have thus been explained. The four French authors whom Dr. Meigs has quoted give the dimensions which we have above mentioned: we may also observe that the same dimensions are given by Naegele, Froriep, and, with the exception of the antero-posterior diameter, by Meckel. It would be curious to ascertain, on what depended the difference in the measurements of the antero-posterior diameter of the brim, by Meckel and by Dr. Burns. The former states it to be four inches four lines French measure, whereas Dr. Burns, in English inches, which are shorter, states it to be only four inches. Our own opinion is, that the correct admeasurement lies between those extremes, and that it is really four inches French measure, as stated by Baudelocque, Boivin, Capuron, Gardien, and the German authorities above alluded to.

2. In speaking of the perineum and fourchette, Dr. Meigs denies that the latter is generally torn during labour; we must confess that we cannot agree with him on this point, and that the fourchette, in spite of all possible care in supporting with the hand, has usually been torn in the first labours. On the other hand, he observes very justly, "Lacerations do not always commence at the fourchette. I have already mentioned a case in which the lower third of the right labium was broken off, and an irregular lacerated wound extended from that point towards the perineum. The accident cannot always be avoided even by the greatest care and skill." (p. 33.) "In other instances, the child has been expelled through a laceration of the perineum proper, not including the fourchette or any part of the vulva, the perforation being made between the anus and vulva." (p. 33.) This latter form of laceration is occasionally though rarely observed, but the rupture of the labium is very rare and appears to result from the unusual rigidity of the perineum, directing the head forwards with so much force against the labia as to produce laceration; we know a case of this sort where a similar injury occurred to the left labium from the firmness with which the perineum was supported, and the unusual smallness of the os externum. The author, in stating this case of ruptured labium, has allowed a little inaccuracy of printing to escape him, which prevents us ascertaining on which side it really took place. At p. 23, he says it was the left, but at p. 33, he alludes to the same case as the *right* labium. In the case to which we have referred it was the *left*, and it would be curious to ascertain whether this was also the left. Would the rupture of the left or right labium depend on whether it was the first or second position of the head; the occiput, in the one case, advancing under the left ramus of the pubic arch, and under the right ramus in the others?

3. We cannot possibly agree with the author in yielding to the long exploded and, we trust, almost forgotten notion, that the gravid uterus when it rises out of the pelvis, "rests on the brim of the pelvis," (p. 34:)

the brim of the pelvis is far enough off the uterus at this time, and has nothing to do with its support.

4. His observations respecting the functions of the vagina are equally objectionable, "it (the vagina) is surely not areolar" (misprinted, we presume, for muscular), "and possesses no other contractility than that which is called elastic, and which is common to the whole of the cellular structure. It closes speedily after the passage of a child, even one of very large size. In some instances where the child's head has lingered long in the vagina, an hour or more elapses before its caliber becomes much contracted; for some hours after the birth of a child, the introduction of the hand into the vagina may be effected with the use of very little force." (p. 35.) Has the author never observed the force with which the placenta or coagula are expelled at moments when the uterus has been perfectly empty and contracted upon itself? Has he never observed the same, even in a more striking manner, when introducing a pessary? Is not the uterus, at term, a muscular organ? and is not the proper or fibrous coat of the vagina a continuation of that of the uterus? Is not the sympathetic action between the vagina and the abdominal muscles, by which they are called into such violent activity when the vagina becomes distended during the last stages of labour, the same as that which exists between them and the rectum? How will he account for the expulsion of an aborted ovum from the vagina? and still more, how could he explain the spontaneous delivery of the head in those (fortunately rare) cases where it has been born and separated from its body and left in utero, if the vagina "possesses no other contractility than that which is called elastic?" The vagina is surely a muscular canal capable of exerting very considerable expulsive power in aid of the uterus, and this has been and is the opinion of all the first anatomists and accoucheurs of Europe.

5. Dr. M.'s remarks on the changes which take place in the ovaries after impregnation, are very incorrect, and betray a greater want of reading on these subjects than we could possibly have expected. "When fecundated, these ovules, called also Graafian vesicles, are removed from the ovary by unknown processes, and, after passing through the canal of one of the Fallopian tubes, are deposited in the cavity of the womb, in order there to undergo the changes that are described under the head of pregnancy. When a Graafian vesicle leaves the ovarium, there remains in that body a yellowish red spot, which from its colour has received the name of corpus luteum." (p. 41.) That it is the ovum which is contained in the Graafian vesicle, and not the Graafian vesicle itself which quits the ovary and passes along the Fallopian tube into the uterus, is a fact so well ascertained as to require no comment from us.

6. The author's remarks, a few pages further on, in his chapter on menstruation, are not more successful. "Writers," he observes, "speak of a greater redness and fulness of the womb and the ovaries, as occurring during the catamenial flow; to all such the question might be very properly addressed, who has seen the womb and ovaries in this predicament?" (p. 49.) We presume that the fact of the uterus and its appendages having been repeatedly seen at these periods, must be well known to most of our readers, and the above remark is the more surprising, since accurate drawings of the appearance which the uterus presents at these periods have been published, and preparations of the uterus, beautifully injected to show the general turgor of its vessels, exist in different museums.

7. Dr. Meigs considers "that cold countries are not so favorable to the increase of population as the milder regions of the south. The reproductive faculty is not so vigorous, where, in consequence of protracted and severe frosts, the productions of the soil are not sufficiently abundant to support an immense population." (p. 54.) This opinion at the first view would appear very plausible, but the result of extensive and accurate observations by no means confirm it. In Iceland the common average of a family is from fifteen to twenty children; in Sweden from eight to ten; in the Netherlands from ten to twelve; in Germany from six to eight; in this country about the same proportion; in France from four to five; in Spain and Italy from two to three; again, however, as we advance further towards the south, especially the northern latitudes of the torrid zone, it increases.*

8. The chapters on Menstruation, Amenorrhœa, and Dysmenorrhœa, are very unsatisfactory. In treating of amenorrhœa, no regular or distinct order of arrangement is pursued; the causes, symptoms, and treatment being so mingled together that it is impossible for the student to form any clear notions upon the subject. The numerous forms under which dysmenorrhœa makes its appearance, and the different periods of the patient's life at which it may come on, are not mentioned, and exposure to "cold and dampness" are the only causes enumerated. We must admit, however, that the author shows himself well acquainted with the disease in its practical details. He very justly observes, "that, where from long-continued dysmenorrhœa the womb has been the seat of a preternatural irritation and affluxion, the organ will in many instances be found tumid and painful. Such a state as this, when once fully ascertained, will throw much light on the nature of the treatment to be adopted under such circumstances as these, which are to be met with far more frequently than is generally conceived of. Rest in a recumbent posture, continued for the space of several weeks, tends greatly to the subduction of the congestive and inflammatory disorder of the womb." (p. 66.) He lays great stress upon rest in the recumbent posture, and observes that, "under this absolute rest great good may be expected from the proper use of bleeding, whether general or local." "These abstractions of blood are followed by great relief, and should be repeated from time to time according to the state of the case, which ought to be noticed by an occasional repetition of the operation of *touching*. During the course just now recommended, the patient should use a purgative or aperient dose every third or fifth day; such as a portion of blue pill and rhubarb, or the sulphate of magnesia. Those days in which no aperient is given should be employed for the administration of antimonials in such form as the physician may select." (p. 67.) Dr. Meigs is in the habit of preferring the precipitated sulphuret of antimony with small portions of camphor, and sometimes morphia or opium. In many cases where the disease is more purely spasmodic, general or local detraction of blood is not required, the sole treatment consisting in allaying the patient's sufferings during the attack by sedatives, and carefully regulating the

* Burdach.

state of the stomach and bowels and improving the tone of the general health during the intervals. We have not employed antimonials in dysmenorrhœa, except in those cases which had been suddenly brought on by exposure to cold, and which were accompanied by considerable febrile excitement. The form of dysmenorrhœa depending on an arthritic or rheumatic diathesis is not mentioned by the author, which surprises us the more, as it was pointed out by his talented countryman Dr. Dewees: the value of the ammoniated tincture of guiacum in this form of the disease is therefore unnoticed.

9. On the subject of leucorrhœa the author's views are simple and practical, although we cannot praise his method of treating it. There is too little attention paid to arrangement. We are apt to ridicule the scholastic and methodical manner in which these subjects are discussed by our German brethren, but it is highly important that a habit should be acquired of following a distinct arrangement; the subject is thereby rendered more complete and more intelligible. Dr. Meigs remarks that "the vast majority of cases are slight, producing even less inconvenience than an ordinary cold in the head; physicians are not told of it, and it disappears spontaneously like other slight catarrhs, leaving the patient in as good health as she enjoyed before the occurrence." (p. 72.) The observation is good; but why has he not extended this comparison to acute leucorrhœa?—a form of disease which is entirely omitted. No notice has been taken of Sir Charles Clarke's valuable observations on the causes of simple leucorrhœa. The effects of gastric derangement on the general tone of the system, more especially in the degree of corrugation which the lining membrane presents, and the connexion between this state and the quantity of mucus secreted, are points which have been so well handled by this author, that we cannot pass over such an omission without expressing our regret at it. The author advocates the use of copaiba, and recommends its being combined with the syrup of tolu, not only to correct the disagreeable taste, but also as conducing to the therapeutical result which is aimed at. His remarks on the utility of bloodletting in this affection would rather stagger a London practitioner:

> "There are few American physicians who would hesitate to employ the lancet for the cure of a rebellious menorrhagia, no matter how feeble the patient might be; and inasmuch as the excretion of puriform mucus is an action depending upon excited vital force, quite equal in intensity to that which causes pure hemorrhage, the use of the lancet appears to be as clearly indicated as a general principle in the one as in the other. It is not necessary to have a hard bounding pulse in order to warrant the use of venesection; a patient who is able to walk across the floor can almost always bear the loss of a portion of blood without great inconvenience, and that inconvenience is generally not to be put in comparison with the invaluable influence of bleeding upon all the emunctories. Bloodletting, therefore, ought to be more freely and commonly resorted to in the management of fluor albus." (p.76.)

This practice may possibly hold good among the healthy inhabitants of Philadelphia, but among a population like that of London, where want of tone and vital energy forms the predominant character of a large portion of the diseases, it would in many cases be absolutely dangerous, and in most very injurious as tending to aggravate that loss of vigour and tone which has been more or less connected with the disease since its first commencement.

10. The ninth chapter on Pregnancy contains much interesting matter, and its value would have been not a little enhanced if the author had omitted all the pages of useless and unprofitable discussion about generation, seminal animalculæ, &c. His observations on the changes which take place in the uterus after impregnation as respects the formation of the decidua and development of the ovum, are very excellent, and it is impossible not to draw a strong comparison between them and the very inferior matter with which the chapter has commenced. Dr. Meigs appears to describe the early development of the decidua from his own observations; and, if this be the case, we cannot but compliment him highly upon his researches in this difficult branch of physiology; as also for the simple, lucid style in which his description is given.

"At the time the act of fecundation takes place, or soon afterwards, the inner surface of the womb undergoes a change which causes it to be covered with a deposit of plastic lymph or albuminous matter which adheres to every part of the inner paries. This deposit has been likened to a coat of white paint plastered over the mucous lining. It might be better compared to the inflammatory exudation of croup, or to those white crusts that are found to line the soft palate and arches in some anginas. Be this as it may, when the small Graafian vesicle reaches the uterine extremity of the Fallopian tube, it finds the orifice closed by this material, which it pushes away as it advances, and at length gets within the uterine cavity; one of its hemispheres resting in contact with the naked surface of the womb, while the other is still resisted by the new deposit which it had thrust away before it as it came into the cavity: it is thus placed betwixt the womb and the deposit. As the new deposit is a mere exudation intended to subserve only temporary purposes, and to be discharged when these purposes have been fulfilled, it is properly called the deciduous coat or caducous coat. But the ovule thrusts the caduca away before it, turns it back, reflexes it, and on that account the decidua has two appellations: 1, decidua vera, meaning all that part that remains in contact with the womb; and, 2, decidua reflexa, or all that part which is reflexed or pushed away by the advancing ovule. When the ovule arrives, it is not so big as a pea, but the cavity of the womb is much greater. The decidua reflexa which closely covers the ovule is just large enough to cover it or inclose it, but the decidua vera is as large as the whole uterine cavity. In process of time the ovule is found large enough to fill up the whole cavity of the womb, and as it always carries the reflexed part of the decidua with it, it follows that the decidua reflexa and the decidua vera come at last to be brought into close contact, and by the continued pressure of the womb are finally so united or fused together as to be indistinguishable from each other." (p. 100.)

We do not make this quotation because it contains anything new, but because we think it gives a clear and correct view of these changes in fewer words than most descriptions with which we are acquainted. With such correct remarks, we are the more surprised that neither Dr. Meigs's reading nor personal observations should have informed him that the Graafian vesicle does not quit the ovary and pass along the Fallopian tube into the uterus. The paragraph which follows the above quotation contains observations on a point of ovology which is entirely new to us. "While the decidua reflexa is thus rapidly increasing in size, pari passu, with the enlargement of the ovum, the space between the two decidua is not void; it is filled with a substance resembling white of egg, contained in a delicate hyaloid membranous network. This substance entirely disappears before the fifth month of gestation." (p. 101.) We cannot presume to offer any remark upon it, not having seen the substance in question, nor do we recollect any description of it among authors on these subjects.

The author's observations on the manner in which the placenta is formed by that portion of the chorion where the ovum is in contact with the uterus are very good, his description is equally so. "Admitting that those villi of the chorion that are in contact with the naked surface of the womb are finally converted into placenta, then the whole of the villi that are in contact with the decidua reflexa never form any union with that substance; the necessity for their existence ceases, and they slowly disappear until the chorion where they covered it becomes a smooth shining membrane." (p. 102.) This fact is well known, and may be demonstrated more or less in almost any ovum which has been expelled at about the tenth or twelfth week. That the ragged vascular prolongations of the chorion during the early periods are "in contact with the naked surface of the womb," we presume there can be no doubt; the ovum at this early period is nourished chiefly by the absorbing power which they evidently possess; they are, in fact, little else than a system of roots for the nourishment of the embryo, being, according to the admirable researches of Lobstein, the extremities of venous branches which unite to form the umbilical vein, the existence of which vessel previous to the umbilical arteries has been confirmed by most original authors on these subjects. "It seems now to be generally agreed that the spongioles of the chorion are not blood-vessels, but merely a sort of areola subserving the purposes of nutritive absorption up to the period when the forces of the embryo acquire a certain degree of development." (p. 103.) The quotation which we have made from Dr. Montgomery's valuable work on the signs of pregnancy in our Number for October 1837, p. 458, bears strongly upon this interesting point; our limits will only permit us to refer our readers to it, as also to our own observations which follow.

11. We can by no means agree with Dr. Meigs that the liquor amnii "can in no manner serve for the nutrition of the child." (p. 105.) The quantity of albuminous matter which it contains during the early periods of pregnancy, and the gradual disappearance of this principle show that this is another means which is designed for the nutrition of the embryo before the placenta has attained its full development and function. Meckel instituted a variety of experiments for this purpose, which prove, we think beyond all doubt, that the liquor amnii holds a very important share in the nourishment of the fœtus, especially during the first half of pregnancy. Neither do we coincide with some other observations in the same page: indeed, they are so unworthy of the excellent remarks to which we have just alluded so favorably, that we would hardly have supposed them to have originated from the same source. The author states that the proper situation of the child "throughout the entire pregnancy is with the head downwards, the breech being towards the fundus uteri. Some cases however occur (he adds) in which the pelvic extremity of the child advances first, and we are bound to believe, when such instances do occur, that the child has been in that attitude during all the latter months of the gestation." (p. 105.) That this is very far from being the case is proved, not only by the different portions of the abdomen in which the movements of the child are felt at different times, but by the different parts of it which are frequently felt presenting when we have the opportunity of examining two or three times before labour; and it is proved still more strikingly by auscultation. His remark in the following page, that "the cervix certainly becomes fuller and larger at a very early period of pregnancy" is very correct; it is to this circumstance that we attribute the common but erroneous notion of the uterus sinking lower into the pelvis at this period, (usually the second month,) and it is to the late Madame La Chapelle that we are indebted for a correct explanation why we can reach the os uteri more easily at this period of pregnancy than at any other.

12. In speaking of Mr. Hunter's views of the placental circulation and giving a short summary of them, the author says "the vessels of the fœtal umbilicus either pump up this fluid (the maternal blood) and take it to the embryo, &c." We cannot allow this to be said "on the authority of Mr. Hunter," whose observations and researches on this subject tend to so opposite a conclusion. We never read his Essay on the structure of the placenta, without feeling more and more thoroughly convinced of its great value: the beautiful simplicity and accuracy of his descriptions; the well-arranged observations, proceeding step by step and leading to such important conclusions, render it almost impossible to add anything further upon this subject. The oftener we read it, or the more elaborate essay on the same subject by Dr. W. Hunter, the more surprised we feel that many authors of the present day should be inclined to doubt the accuracy of such observations.

13. Dr. Meigs's attempt to explain the source of hemorrhage when the placenta has been separated from the uterus (p. 115) is exceedingly confused and incorrect; he supposes it to proceed from what he calls "hemorrhagic irritation;" and appears to have no notion of its coming from the mouths of those arteries whose communication with the placental cells had been destroyed. His views respecting the action of cold in uterine hemorrhage of this description is equally incorrect; he supposes it to act as in epistaxis, hæmoptoe, or hæmatemesis! His treatment of the whole of this subject is very imperfect, and betrays but little acquaintance with the literature of it, although it has been enriched by the observations of Wrisberg, Meckel, Lobstein, and the Hunters,—not to mention the interesting papers in the Medical Gazette by the late Dr. Hugh Ley, and Messrs. Stanley and Mayo.

14. The remarks on the treatment of abortion are simple and practical; and we were much pleased to notice some observations on the subject of retained ovum which interested us much and with which we fully concur.

"A good many cases of abortion in the early stage, as from the sixth to the tenth week, have fallen under my notice in which the uterus was unable to expel the remains of the ovum, and in which I could not extract it. The female in such instances has always recovered without the ovum having been visibly discharged, but there was always an excretion continued for many days of offensive dark-coloured grumes and sanies which I accounted for by supposing that the substances in the uterus had macerated and come off in a state of semi-solution. I think that there is no danger in leaving such occurrences in the hands of nature, and that it is better to do so than to reiterate attempts to extract by force that have perhaps already proved quite vain, especially considering that there is as great danger of exciting inflammation by those attempts, as could be anticipated from the gradual maceration of the ovum. I am not disposed to deny that the presence of a putrefying substance even of a small size in the womb is capable of developing violent inflammation and fever; but it has not happened so with me, and I have given the same opinion to some medical friends, by whom I have been consulted, without the least cause to regret having given such advice. Let me be clearly understood, however, to recommend that the last remainders of the ovum should be brought off where it is practicable by means of any reasonable efforts." (p. 130.)

We have had repeated opportunities of proving this fact, although we must confess that as far as respects portions of placenta which have been retained after labour at the full term, the discharge has not been as the author describes, but to all appearances perfectly natural lochia, except that it was rather sparing in point of quantity. We quite agree with him that "the presence of a putrefying substance, even of small size, is capable of developing violent inflammation and fever;" and we have every reason to suppose that the absence of these formidable symptoms in cases of this sort has been owing to the full and firm contractions of the uterus effectually excluding all excess of external air.

15. We scarcely understand why Dr. Meigs experiences such difficulty in accounting for the symptoms produced by prolapsus uteri. Both the mechanical and constitutional symptoms are in our opinion exceedingly easy of explanation; and we should be inclined to attribute a portion of the difficulty of which he complains to the wrong principle upon which he sets out, viz. in denying that the vagina has anything to do with supporting the uterus. The excellent observations of Dr. Dewees would, we conceive, have been sufficient to show that this was very far from being the case. We are the more surprised at his observations, because, when he speaks of employing a pessary, he observes that "the pressure which it must exert upon the tissues which inclose it, could not fail to have a great tendency to condense those tissues, and thus obviate the relaxation of them which constitutes the pathological essence of the disorder." (p. 138.) No notice whatever is taken of the utero-abdominal supporter invented by the late Dr. Hull, we believe, of New York. We should not have ventured to allude to it upon the present occasion had not the author entered pretty fully upon the consideration of prolapsus, merely because a woman may be liable to it if she leaves her bed too soon after miscarriage, the chapter itself being headed "pregnancy." Dr. Hull's instrument acts chiefly by removing the weight of the intestines, and from the lower part of the abdomen and pelvis by means of a very ingeniously contrived truss, which, by pressure carefully applied in a direction upwards and backwards, effectually prevents their gravitating upon the uterus and forcing it down: we have tried it, and we must confess with a preconviction of its inutility; but it answered its object most satisfactorily—the patient was immediately and agreeably relieved from the painful sense of weight and bearing down from which she had hitherto suffered.

16. We have observed in a former Number of this Journal, that the condition of the child, as respects its state of nourishment, is in no wise proportionate to the condition of the mother; that stout hearty women frequently bear meager ill-nourished children, whereas those who are emaciated by disease or want are frequently observed to have remarkably stout children. Dr. Meigs's experience perfectly accords with our own.

"I have witnessed several accouchements in females reduced to the last degree of weakness and emaciation by pulmonary consumption. In these cases I have not found that the child partook of the debility and cachexy of the unhappy mother. In one case that fell under my care the patient laboured under laryngeal consumption, and was to the last degree emaciated and feeble, notwithstanding which her pregnancy held out until the completion of her term, which she survived only four or five days. Her child was excessively fat, weighing above ten pounds, and has continued to enjoy excellent health up to the present time, when it is between five and six years of age. These instances convince me that no methods that have yet been discovered can serve to prevent the development of the child, and thus cause the woman to suffer less in her accouchement than she would do if her child were to be small." (p. 147.)

17. Dr. Meigs follows chiefly the French authors in describing the various presentations of the child, and the manner in which they advance through the passages; he applies the term vertex to the posterior fontanelle, and therefore, until the reader has understood this peculiar meaning, it is difficult to understand him. The whole chapter is very theoretical, and bears the marks of more ingenuity than of careful and practised examination; if this had not been the case we should have seen fewer such expressions as "dip of the child's head," "pivot motion," "rotation," &c. He speaks of the child being in danger of strangulation, (p. 182,) if the cord be tight round its neck when the head is passing over the perineum. Does the face in this position swell and grow livid from strangulation? or can such a term be applied to a child which has never used its lungs? Mauriceau long ago showed correctly the cause of its death, viz. from an apoplectic state of the brain being induced by the pressure of the cord upon the neck; it has nothing to do with strangulation. In other respects his remarks are simple and practical. In the page just referred to he observes, " the young practitioner and the student should be warned against falling into a habit of beginning too early to support the perineum. If the part be too early pressed upon with a napkin it might become heated and thus lose its disposition to dilate," and in the following page he observes, that where the shoulders have passed, " it is considered bad practice to drag out the body except in very particular instances: the womb and abdominal muscles are sufficiently powerful for that object; and if it be permitted to come away slowly we shall have a more complete contraction of the womb, and a more ready detachment and extension of the placenta." (p. 183.) His remarks on the management of the placenta are equally good; he directs that it should be turned round several times at the moment of its expulsion, so as to twist the membranes into a cord and thus ensure their removal without laceration.

"The influence of position in determining the momentum of blood in the vessels is well known to the profession; but there are few cases where it is of more consequence to pay a profound regard to this influence than in parturient women. A uterus may be a good deal relaxed or atonic, and yet not bleed if the woman lie still with the head low, whereas upon sitting up suddenly such is the rush of blood down the column of the aorta, the hypogastrics, and the uterine and spermatic arteries, that the resistance afforded by a feeble contraction is instantly overthrown, the volumes of blood escape with an almost unrestrained impetuosity. The vessels of the brain under such circumstances become rapidly drained, and the patient falls back in a state of syncope which now and then proves immediately fatal." (p. 192.)

Dr. Meigs very properly insists on the danger of allowing coagula to remain in the uterus, as tending to favour internal hemorrhage. He observes that " strong uteri never permit them, weaker ones allow considerably larger ones to be formed, and very feeble wombs fill until the woman faints or dies." (p. 195.) After so much valuable and practical matter we regret that he should again theorise. Obliquity of the womb is surely no cause of face presentations: we meet with them where the uterus is perfectly straight, and we constantly see cases where the fundus is very much to one side and yet the child presents as usual. We fully agree with the author, however, in saying that there are only "two positions of the face, one with the chin to the left and one with it to the right of the pelvis," (p. 204;) but we would rather invert the order of these two positions, and say with Professor Naegele that those face presentations with the chin to the right are the most common. Dr. Meigs very properly differs from the common rule of examining during a pain: "there is no need for pressing against the bag of waters during the pain, because by waiting until the pain subsides, the bag becomes relaxed, and can then be pushed back again within the mouth of the womb so as to enable the finger to touch the head." (p. 219.) We cannot, however, agree with the author in stating that "the length of the gravid uterus at full term does not exceed twelve inches," (p. 228;) the fundus is well known to have reached the umbilicus at the sixth month, and this in a moderate-sized woman would be little short of twelve inches, whereas at the full term the fundus occupies the scrobiculus cordis.

It is satisfactory to see how clear and correct our author is on all points of practice. When speaking of breech presentations and the danger to which the child is exposed from pressure upon the cord when the head is passing through the pelvis, he adds, "It is my unfailing custom therein to order my forceps to be put in readiness as soon as I ascertain that the presentation is not one of the head, and I feel very well assured that such a precaution if generally observed would preserve many a life which is now lost, either by delay in the delivery of the head, or by pernicious attempts to extract by pulling at the neck, to which the temptation is so strong in moments of great anxiety for parent and offspring." (p. 238.) This is very excellent practice, and we have no doubt that if the forceps was at hand in every case of turning or breech presentation, many more children would be born alive under such circumstances than is the case at present. The valuable observations on this subject by Professor Busch, of Berlin, of which we have given a very brief notice in our tenth Number are well deserving of notice. By attention to this point Professor B. has succeeded in saving the child's life in forty-one turning cases out of forty-four, a degree of success in this operation which has never been even approached by any practitioner. Dr. Meigs entertains the old and long-disproved notion respecting obliquity of the uterus being a cause of the child presenting unnaturally: it will be scarcely necessary to discuss the point, the highest authorities both of former as well as modern times combining to reject it entirely. His views respecting arm presentations are very unsatisfactory: he remarks that, "as there are two shoulders, a right and left one, there must be a set of positions for each shoulder." (p. 247.) Of what use in practice is all this complication? We pass our hand along the anterior surface of the child if possible, in order to reach the feet, and if the arm be prolapsed the direction of the hand will generally guide us. If the case be at all difficult we must direct the hand wherever there is most room. His directions for introducing the hand are excellent, as indeed his observations generally are on subjects purely practical.

In enumerating the various signs indicative of a shoulder presentation, the author omits the important one of not being able to feel any presenting part at all. The os uteri has gradually dilated, a large bag of liquor amnii (not always deviating from the regular form as the author mentions) is felt, and still no presenting part has descended within reach; this is a very suspicious sign, and in a primipara is a bad one, because the head is known to be usually so low in the pelvis at this time. We are far from considering this an infallible sign, however, because the breech may present, and this is generally very high up in the pelvis at the commencement of labour; or it may be a contracted brim preventing the head from descending; still nevertheless it is a symptom which ought never to be overlooked. Dr. Merriman's admirable observations on this point are well worthy of attention.

The author's directions as to the precise moment for introducing the hand in turning are excellent: "during a pain, two fingers, and then three, of the left hand should be passed into the vagina, &c." (p. 250.) The patient is much less sensible to the introduction of the hand during a pain, and we thus in a great measure diminish what is the most painful step of the operation; the hand, moreover, is now ready to enter the uterus the moment the pain goes off. "It is always desirable to get the hand out of the uterus as soon as may be; and it is far better to turn by one foot or by a knee than to incur the risk of lacerations or contusions of the organ by a tedious search after the other foot, which, if it be not originally near its fellow, is very hard to be found by any search after it." (p. 251.) This advice is important in many respects, wherever turning is at all difficult, or has to be performed under unfavorable circumstances. We are rarely able to bring down both feet at first, except where, by some very lucky chance, they are lying close to each other. Generally speaking, by the time we have brought one leg fairly into the vagina, the position of the child is beginning to change so materially that the knee or foot of the other lower extremity will come within reach. We hold it to be highly desirable to bring down both feet, if possible, as there is no doubt that the nates of the child enter the brim of the mother's pelvis much more readily. We cannot agree with the author in a sentence which follows immediately after. "Having found the foot, if a pain comes on immediately, and becomes a severe one, the foot should be let go, and caught again after the pain is gone off, according to the discretion of the operator." (p. 251.) Not only do we run a chance of the foot getting quite out of reach when the pain has ceased, which is of no slight consequence, but we are turning the pain itself (now that we have reached the foot) to no account. Our practice, wherever the foot is high and difficult to reach, is to hold it quite still for a pain or two; feeling assured that, as soon as the pain is over, it will have come a little more within reach, and enable us to take a firmer grasp against the approach of the next pain. The pressure of the other hand upon the abdomen is very important, and, as Dr. Meigs truly observes, "ought not to be neglected." He gives us no directions as regards the membranes in turning: whether we are to rupture them or insinuate the hand between them and the uterus; a point of practice which is of considerable importance, and upon which it would have given us much pleasure to have known his opinions. We presume that he recommends the latter mode,

CHARITY HOSPITAL, MEDICAL LIBRARY.

as this is evidently his practice in cases of placenta prævia: having introduced the hand between the placenta and uterus, he observes, "the membranes may then be ruptured high up in the uterus, and the feet immediately sought for." (p. 264.)

Dr. Meigs denies that the forceps act by compression as well as by extraction; but here we must differ from him: there can be no doubt that a degree of pressure, carefully applied, does alter the dimensions of the fœtal head, and assists greatly in adapting it to the passage through which it has to come. Baudelocque's experiments on the heads of still-born children prove nothing as to the present question: indeed, their fallacy is shown by the alteration of shape which the head presents immediately after labour in most children, especially where it has been the mother's first pregnancy. The author's directions for applying the forceps are pretty nearly those of the French school, the patient being placed on her back for the operation. They are minute in some respects, but incomplete and meager in others. His precautions in extracting the head are good; but, as we cannot agree with him in asserting that the forceps do not act by compression as well as by traction, we consider his objection to keeping up a continued pressure upon the head, either by grasping or tying the handles, as unfounded. We have ever found in difficult forceps cases, where the head was considerably impacted, that our only chance of avoiding perforation was to maintain a firm but steady pressure upon the head by the above means, until it had so elongated and adapted itself to the passages as to produce less obstruction to its advance: the pressure thus artificially applied is surely as close an imitation of nature as using extractive force is: we are, in fact, only using the same means to alter the shape of the head which she avails herself of.

18. In speaking of puerperal fever, the author observes, "the peritoneal coat of the womb is greatly expanded or stretched in the last stages of pregnancy. The broad ligaments are drawn up on the sides of the uterus to a considerable height, while the portion of the membrane that lines the front and sides of the belly is also put greatly on the stretch. This tension could not but increase its natural proneness to take on inflammatory action, if exciting causes should be applied after delivery," (p. 246;) and yet he commences the following page with a remark which appears to us rather inconsistent: "The relaxation of the peritoneal membrane that follows delivery, and the reduction of the womb to a small size, is, beyond doubt, one of the most fruitful sources of inflammation of the membrane." (p. 347.) The author's high encomium on bleeding in puerperal fever, as well as his observations under this head, are interesting, inasmuch as they show us under what form the disease has most frequently manifested itself in Philadelphia.

"I would earnestly endeavour to impress upon the mind of the student of medicine the vital importance of great promptitude in his attention to the earliest signs of this dreadful malady. I would convince him that the principal feature in the disease called childbed fever is peritonitis; that the inflammation is so acute, and the tissue in which it is seated is so inflammatory, that the malady is capable of hurrying through its curable stages more rapidly than even the redoubtable croup; and, what is of still greater moment, that it is in the incipient stages nearly as curable as croup, and that the remedy (or I might say the cure) consists in the bold and judicious employment of venesection. Let me ask what can be the value of any remedy short of venesection in a malady like this, which presents a case of pure inflammation, occupying, or making haste to occupy, not a few square inches, but many square feet, of a membrane that serves as the investment of the most important organs?" (p. 352.)

Would that puerperal fever always appeared in this manageable form! Many are the times when we would have hailed the appearance of peritonitic symptoms as a fortunate occurrence for the patient. In some of the very worst cases that have fallen under our notice, there was not one symptom of inflammation during life, or one mark of it to be discovered after death: some of these, from the first perceivable change in the patient until the fatal termination of the disease, have run their course in twenty-three hours. We must, however, refer the reader to our reviews of the valuable works of Mr. Moore and Dr. Ferguson on Puerperal Fever, (vol. II. p. 481, and vol. VII. p. 482,) for our opinions on this momentous subject.

Dr. Meigs's treatment in the form of puerperal fever which he has described is very judicious; and the manner in which he has pictured the last sad hours of an unsuccessful case are so graphic and true that we must be allowed to make one more quotation.

"To find an improvement in the patient's ability to move herself, with a corresponding improvement in the circulation, is of the most favorable augury; but, to observe the pulse increasing in frequency while it also becomes more feeble, with diminished heat of the members and augmented heat of the body,—to discover a disposition to singultus, with an eructation of fluids into the mouth, an anxious expression of countenance, high and frequent respiration, with increased ability to move the legs, and diminished pain on pressure, are all indicative of the cessation of inflammation of the peritoneum; but it has ceased not by resolution or a return to health: it has come to one of its natural terms in effusion. The inflammation is at an end, and the patient begins to die. It would seem that the forces of the living economy have exhausted themselves in the struggle with a malady, which, though they conquer it at last, yet are themselves destroyed in the moment of victory. There soon comes on a vomiting, or rather a violent eructation or gurgitation of dark-looking fluid; the patient mutters, she picks the bedclothes, she clutches at muscæ volitantes; the diaphragm labours to carry on, in vain, the work of respiration; the hands and feet acquire a livid hue and are clammy; the pulse becomes a thread, it ceases in the wrists, and she dies probably in the act of regurgitating from the stomach the last draught which the anxious hand of friendship or love has tendered as a solace or as a hope." (p. 358-9.)

The work terminates with a few observations on the Morbus cæruleus. The author thinks that permanent relief may be given by placing the child upon its right side, propped up by pillows at an angle of thirty degrees; that, by thus "maintaining the heart in such an attitude, the left auricle would be perpendicularly above the right one; and that the effect of gravity alone on the blood would gradually operate in such a manner as to allow it to flow off into the ventricle, instead of finding its way to the systemic auricle." (p. 367.)

We here close our review of Dr. Meigs's book. It decidedly contains much sound practical information, but still we cannot place it in the slightest competition with Dr. Dewees's admirable work. The literature is everywhere too defective, and, by apparently confining his reading very much to the French authors, and those (with the exception of Mauriceau) chiefly of the present day, he has been too frequently led into the vague theoretical views in which they are so apt to indulge.

II. Dr. MARTIN's work consists of a collection of papers upon subjects more or less connected with obstetric practice, which are evidently the result of much experience gathered from long and extensive observation. If we overlook the foolish and unworthy habit which our Gallic neighbours are so apt to fall into, of indirectly arrogating to themselves the whole merit of certain facts and modes of practice, which, however good and praiseworthy, had been known in other countries long before them, we shall find that M. Martin's work contains much that is valuable and deserving of attention. The work itself, as the preface informs us, was originally a septennial report of cases at the Charité Hospital of Lyons; the author's remarks are consequently short and practical; and we shall therefore in noticing it, confine ourselves, as far as possible, to giving short extracts here and there of whatever we may consider useful or interesting to our readers.

1. In speaking of *abortion*, the author observes that "the most frequent causes are mental emotions and inveterate chronic venereal affections; under the last-mentioned circumstances, I have almost always found the products of conception macerated and as it were decomposed, without any fætor, at least in those cases where no access of the external air had taken place from rupture of the membranes. It is so true, that atmospheric air is a necessary agent to putrefactive fermentation, that I have known a full-grown living fœtus born, holding in its left arm a macerated withered abortion, without any smell, which from its size had evidently lived to the fifth month." (p. 6.)

2. The ages at which the female system becomes susceptible of impregnation, and ceases to be so, vary exceedingly. Dr. M. has met with a case of pregnancy at the age of thirteen years and a half, and another where the patient was somewhat older, but had not as yet menstruated. On the other hand, he mentions having once attended a patient in labour at the age of fifty-four, whose appearance was as decrepid as that of most women at sixty. We have ourselves met with cases of pregnancy at this extremely early age, but do not recollect one where the menses had not already appeared. The fact, however, of impregnation taking place before the appearance of the catamenia has been noticed long ago by Levret. A case of pregnancy at so late a period of life as that described by the author, we must confess we have not met with.

3. In describing the effects of difficult labours, M. M. remarks that "when the capsules of the pelvic symphyses had been torn, a species of ligamentous callus formed behind the pubes, which rendered the succeeding labours more difficult. I have seen collections of matter follow laceration of the pubic and sacral symphyses, the result of which has been usually fatal." (p. 16.)

4. In cases where the child is coming into the world with the feet foremost, and the os uteri has contracted spasmodically round its neck and thus retards the delivery of the head, the author has found the application of cold water directly to the os uteri, to have the effect almost invariably of removing this spasmodic condition of its fibres, and thus facilitating the expulsion of the head. We have no experience in the employment of such a remedy in these cases, but from what we have observed of the effects of cold injections into the vagina in dangerous cases of hemorrhage after labour, we should have expected a very opposite effect.

5. M. Martin makes the discovery that wherever he has ventured to apply the forceps, the small diameter of the pelvis being less than three inches, that the child was born dead! We should have been inclined to ask whether the mother did not die also? We much question also the correctness of his observation where he fixes two inches and three quarters as the limit within which the Cæsarean operation is required; as numerous cases prove that, even with a greater degree of contraction, the child can be delivered by perforation and embryotomy.

6. We fully agree with the author in the following remark: "The adhesion of the placenta to the uterus has appeared to me to arise from a morbid condition of the internal surface of this organ, for I have rarely observed it in one confinement without meeting it in the succeeding ones. I have seen it four times successively in the same individual." (p. 20.) It is difficult, nay perhaps impossible, to say what is the condition upon which this state depends; and the opportunities of examining the nature of this adhesion minutely are very few. In some cases, where the attachment is exceedingly firm, the whole substance of the placenta breaks up in the attempt to remove it, becoming a soft ragged fibrous mass which adheres with the utmost tenacity to the uterus.

7. We might have added some critical observations on the author's views of placenta prævia; but our object, in the present instance, is rather to give a brief yet comprehensive account of whatever is good and useful in a work which contains so much valuable matter, and which has evidently been the result of much observation. We therefore pass on to an excellent remark on the subject of epidemic puerperal fever. "It follows (says M. M.) the type of the prevailing epidemic, and usually takes the same character." (p. 27.) The correctness of this observation has been strikingly confirmed during the epidemic of malignant typhus which has lately prevailed so destructively in London. In several puerperal cases which we have witnessed, the livid, rubeoloid eruption was precisely similar to what was so constantly observed in patients of both sexes who were at that time suffering from the prevailing disorder: in many cases the patient seemed suddenly struck down by a deadly influence, against the overwhelming effects of which, the powers of life appeared capable of offering no resistance.

8. On the subject of stillborn children he says, "Some of them present all the appearances of death, because the lungs have not yet acted, a state which may be called asphyxia; others arise from the pressure which the brain has undergone from the gorged state of the cerebral vessels." (p. 34.) We do not quote this as an original observation, because this cause of asphyxia has been pointed out long ago by Smellie and more lately by Chaussier, but merely from a wish to call the attention of our junior readers to a fact which perhaps has not received sufficient notice. His treatment of asphyxia neonatorum is good, but presents nothing new, except that he recommends plunging the child into a bath of hot wine, which would be rather an expensive remedy in this country, and which cannot have one great advantage which hot water possesses, viz. of a large quantity being kept hot in readiness the moment that the child is born; dipping the child for an instant into hot water which is at too high a temperature for it to remain in, and then suddenly exposing it to the air and again repeating the process is a plan which has, we believe, been used with considerable success by the matron at one of the Lying-in

Hospitals of London. "Bleeding from the cord," adds M. Martin, "has always sufficed to relieve the brain and remove the apoplectic condition. I can assert from my observations that the appearances of death in new-born children are very deceptive. I have recovered by perseverance children which presented every sign of being dead. I met with one case among others, which after having exercised my patience for two whole hours, recovered at the moment I was about to desist from my attempts. I have seen many children die who had continued to cry loudly from the moment of their birth, and yet upon the most careful examination after death no cause could be detected upon which the slightest conjecture could be hazarded." (p. 35.)

9. Dr. Martin considers that he has frequently prevented infection being communicated to a child from a syphilitic mother, by throwing up oleaginous injections into the vagina both before and during labour. This, of course, applies chiefly to where the mother has been but recently infected, and not to old cases of long standing where the whole system has been pervaded with the disease.

10. In his treatment of Nævus, he never uses a cutting instrument; but where it is situated upon a part which offers a sufficient degree of resistance to admit of compression, he applies it by means of a pad which is fixed by a circular spring upon the head: by this means he informs us that he has occasionally succeeded in removing the nævus, and where he has failed in obtaining this result, he has at any rate been able to arrest the progress of its growth.

11. Dr. Martin describes a case of hydrocephalus in a child eighteen months old where he performed paracentesis capitis, and drew off a pint of serous fluid; for a short time after, the child appeared much relieved, but died on the fifth day. The date of this case would have been desirable, as it would have shown whether he had performed this operation before or after it had been tried by Dr. Conquest in London, to whose observations however he makes no reference.

12. He mentions a case of hydrophobia where the disease was prevented, by cauterizing the wounds deeply with the butter of antimony; the patient, a girl, had been bitten by a mad wolf which had attacked several people, many of whom died from hydrophobia. We remember seeing a somewhat similar mode of treatment adopted by the celebrated Rust, of Berlin: he premised, however, a very effectual excision and dressed the wound afterwards with liquor potassæ, a remedy which he considered to possess a peculiar power in decomposing animal poisons. How strikingly has this latter opinion been confirmed by the masterly researches of Dr. Stevens.

13. M. Martin has given some very interesting observations on the changes which the fœtus undergoes when it dies in utero. He divides them under two heads, firstly, where the fœtus has died in utero without rupture of the membranes; and secondly, where it passes rapidly into putrefaction, where the os uteri being open and the membranes ruptured it has come into contact with the atmospheric air. Of the first class he has given six cases of abortion at an early period, in all of which the ovum was expelled entire, but without any traces of the embryo. This disappearance of the embryo after its death in the ovum has been noticed by many authors, more especially by Smellie, who considers that this dissolution of the embryo may take place in the course of a few hours where pregnancy has not advanced beyond the month, and even in the second month twenty-four hours will be sufficient to effect this change. What the precise nature of this process is, we cannot venture to determine; but experience shows that after such a change the ovum seldom, if ever, remains any period of time in the uterus without undergoing considerable alterations of structure, which are chiefly confined to the chorion: this becomes much thicker, grows with rapidity, and assumes the peculiar fleshy structure which gives the ovum the name of *mole.* M. Martin has limited the above-mentioned cases to the first two months of pregnancy, and as regards the disappearance of the embryo he is perhaps right; but it is very hazardous to draw such lines of demarcation; we regret that the condition of the membranes was not more precisely noticed, as we cannot but think that some of those changes in them to which we have just alluded, would have been observed. It may be, perhaps, a question whether the membranes of the ovum would undergo those changes which constitute a molar growth in cases where the embryo has perished without any further injury having been sustained by the ovum; for it would seem, according to the admirable observations of Dr. Simpson of Edinburgh, that sanguineous extravasation to a certain extent is necessary to produce the fibrous lobulated appearance so frequently observed in abortions of this character. Several interesting cases of twin-pregnancy are also given, where one fœtus had died whilst the other went on to be developed up to the full time. He also mentions an extraordinary instance where expulsion took place *during sleep* at the beginning of the seventh month, the fœtus having evidently died in the sixth month. In summing up his remarks on these subjects, the author observes as follows:

"The fœtus which dies in utero may remain there a considerable time without passing into putrefactive decomposition, so long as the os uteri is closed and the membranes unruptured. The changes which a dead fœtus undergoes in the uterus vary according to the period of pregnancy at which it ceased to live; thus, in the early grades of its development, when its structure presents but little more than a gelatinous consistence, it is dissolved in the liquor amnii which becomes thick and mucilaginous. We can find no traces of the embryo in the cavity of the membranes which may continue to remain in the uterus for some time. At a more advanced stage, viz. from the second to the fifth month it withers and shrivels up; it resembles rather a little mummy of a yellow colour, or as if it had been kept for some time in spirits. The membranes frequently undergo a similar change, the liquor amnii disappears, and in its place we find a thick and almost earthy fluid with which the fœtus becomes incrusted. From the fifth month to the full term of utero-gestation, the fœtus macerates, increases in size, and becomes soft and friable; the epidermis separates, forming phlyctenæ of more or less extent; the skin becomes discoloured, as also the muscles, which tear with the slightest effort; although animal decomposition is evidently commencing yet there is merely a faint sickly smell, and nothing of that powerful fetor which characterizes putrid fermentation." (p. 95-6.)

14. Dr. Martin has devoted a chapter to the subject of retroversion of the uterus, and has collected an unusual number of cases of this rare displacement. Two of them are peculiarly interesting. The one (p. 156), occurred on the sixth day *after delivery* in consequence of the patient making a sudden effort to save one of her children who had tumbled down in playing at her bedside: the uterus was replaced after some difficulty and the patient recovered. The other (p. 161) is interesting from having been treated by puncture. The uterus had resisted every attempt to replace it; and the practitioner having felt a distinct fluctuation through the rectum, a curved trocar was introduced about five or six lines up the intestine at the spot where the fluctuation was most evident; a quantity of limpid serous fluid escaped, which diminished the size of the tumour in the vagina and relieved the patient's sufferings. Pains came on three days after the operation, and on the following morning expelled a fœtus of about four months and a half. The patient did well.

15. In the chapter *on Inversion*, M. Martin also gives a considerable number of cases. The third is peculiarly interesting from the operator having diminished the size of the uterus by compression before attempting to return it. This mode of practice was adopted with success by the celebrated C. White, of Manchester, in a case of unusual difficulty, and described by him in his work on Lying-in Women, a point which ought to have been noticed by the author. M. M. considers that *inertia uteri* is one of the great predisposing causes of inversion. "From the moment that the fundus begins to pass through the os uteri, the patient experiences a violent impulse to bear down, with painful tenesmus, producing contraction of the abdominal muscles which gradually push down the fundus; in proportion as this approaches the vulva, a painful sensation of weight is felt in the pelvis, and the patient complains of severe pains in the kidneys and groins resulting from the ligaments of the uterus being put so forcibly on the stretch." (p. 212.) He describes an exceedingly interesting case of chronic inversion of two years standing, and where the powers were gradually breaking up under the constant discharge of bloody mucus from the tumour and profuse hemorrhage at the catamenial periods; the patient had consulted the celebrated Leroux, of Dijon, who recommended a palliative treatment; her medical advisers at Lyons proposed the ligature, which was accordingly applied; the symptoms were but slight, the uterus came away on the nineteenth day without any hemorrhage and with complete restoration of her health: the lady was alive in 1833, forty-two years after the operation. The whole chapter contains much interesting information, although not much of it is original; the literature, as elsewhere throughout the work, is extremely defective.

16. The next chapter is on imperforate os uteri, a condition which has lately been made the subject of much interesting discussion in this country. His third case is quoted from Lauverjat: the patient was a primipara; a tumour was found filling the vagina and protruding during the pains: after careful examination he felt convinced that this tumour was formed by the cervix uteri, the orifice of which was entirely obliterated; he made an incision into the tumour through which the child was safely delivered: after labour the os uteri was sought for in vain, nor was it found in its natural condition until two months after. In a similar case which occurred to the author himself, a slight depression was detected, after minute examination, in the vicinity of the left sacro-iliac symphysis; after a short interval, the patient having had some very violent pains, he again examined and found the os uteri somewhat dilated at the spot where he had felt the depression: he supported the tumour whilst he pulled the os uteri forwards into the middle of the vagina, and the labour proceeded naturally and successfully. "The analogy," says M. M., "between this case and the one related by Lauverjat is striking, and leads us to suspect, 1st, that this celebrated accoucheur made an incision into the anterior portion of the uterus; 2d, that the os uteri, which in this case was found two months after delivery to be perfectly uninjured, had been situated very high up and far backwards." (p. 245.)

17. The chapter on deposits of pus in the appendages of the uterus in consequence of labour, is very interesting. In some cases the author has succeeded in checking puerperal inflammation by the application of leeches to the labia, a mode of practice which has recently been improved upon by a distinguished accoucheur in the Irish metropolis, and attended with great success. Where the symptoms indicate the formation of pus, Dr. Martin uses the caustic potass, for the double purpose of producing a firm adhesion between the parietes of the sac and of the abdomen, and of obtaining an exit for the confined matter, which latter object he sometimes expedites by incision where the parietes are very thick.

18. The author's other chapters, although for the most part interesting, do not contain anything new or of sufficient interest to require notice here. The last chapter, on the history and application of the forceps, is of considerable length. The history is very meager and faulty and full of misspelt proper names for which the French authors are so notorious. M.M. describes a peculiar species of forceps which appears to be generally used in Lyons, the blades of which do not cross, but, as far as we can understand from the description, they cannot be looked upon as an improvement.

We take our leave of Dr. Martin's work with feelings of great respect for its industrious and experienced author. It contains much valuable matter, and will prove a useful work of reference both to the practitioners and the author.

---

